# Announcement: ICCC34 - 34th International Conference on Coordination Chemistry

**DOI:** 10.1155/MBD.1999.369

**Published:** 1999


					ICCC34
ON
34th INTERNATIONAL CONFERENCE
COORDINATION CHEMISTRY
9-14 July 2000, Edinburgh, UK
This will be the 'Golden Jubilee' meeting dedicated to Joseph Chatt, founder of the ICCC
Conference Topics
Chemistry of Life
Structure and Dynamics
Biotechnology & Medicine
Technological Advances
21st Century Materials
Plenary Speakers
Professor L Fabbrizzi (Pavia, Italy)
Professor O Kahn (Bordeaux, France)
Professor M Poliakoff (Nottingham, UK)
Professor K N Raymond (Berkeley, USA)
Professor H Schmidbaur (Munich, Germany)
Professor S Shinkai (Kyushu, Japan)
Professor L M Venanzi (Zurich, Switzerland)
Professor S B Wild (Canberra, Australia)
RSC Joseph Chatt Lecturer
Professor C D Garner (Manchester, UK)
Scientific Committee
Professor P J Sadler (Chairman)
Professor A J Welch
Professor D E Fenton
Professor D W Bruce
Professor C E Housecroft
Professor R E Mulvey
Professor D J Cole-Hamilton
Professor R W Hay
Professor S K Chapman
Professor D M P Mingos
Professor T Vlcek
Dr S Parsons
Professor A K Powell
Dr M Ward
Dr N Winterton
Organising Committee
Professor P A Tasker (Chairman)
Professor D J Cole-Hamilton
Mrs E R Watt
Professor C E Housecroft
Dr J F Gibson
Professor R W Hay
Professor B F G Johnson
Professor P J Sadler
Professor P O'Brien
Professor A J Welch
Dr L J Yellowlees
Professor J J Monaghan
Further Information
Further information on this meeting can also be found at:
http:l/www.ed.ac.ukl~euchem/ICCC3411CCC34.html
http://www.rsc.org/lap/confs/iccc34, htm
For further details of the ICCC34 conference by mail, please contact:
Nicola Durkan
ICCC34, Royal Society of Chemistry
Burlington House, London, UK WlV 0BN
fax :+44 (0)171 734 1227
Email" Conferences@rsc.org (Please use subject header "1CCC34")
369

				

